# Vegetarian diet and pregnancy outcome

**DOI:** 10.1016/j.eurox.2024.100345

**Published:** 2024-10-09

**Authors:** Johanna Kristiina Reijonen, Kati Maaria Hannele Tihtonen, Tiina Hannele Luukkaala, Jukka Tapio Uotila

**Affiliations:** a Department of Obstetrics and Gynecology, Tampere University Hospital, Wellbeing Services county of Pirkanmaa, Finland; b Faculty of Medicine and Health Technology, Tampere University, Finland; c Tampere University Hospital, Research, Development, and Innovation Center, Finland; d Tampere University, Faculty of Health Sciences, Finland

**Keywords:** Vegetarian diet, Omnivores, Pregnancy, Pregnancy outcome

## Abstract

**Objective:**

Vegetarian diets are becoming increasingly popular. Our aim was to evaluate the association of a vegetarian diet with pregnancy, labor, and newborn’s outcome.

**Study design:**

This retrospective case-control study included 150 women on a vegetarian diet and 300 omnivores. The data were collected from Tampere University Hospital pregnancy database from January 2015 to April 2021. Diet was a self-reported variable. Outcomes of interest were compared between the groups. The frequency of small-for-gestational age (SGA) newborns and low birth weight were primary outcomes. Other parameters concerning pregnancy, labor, and newborn served as secondary outcomes.

**Results:**

The rate of SGA at the 10 % cutoff was lower in the vegetarian group. Based on a definition of two standard deviations, SGA did not differ between the vegetarian diet group and the control group. The median birthweight was significantly higher in the vegetarian group. Gestational diabetes (GDM) was more common in the vegetarian group, however the numbers of large for gestational age (LGA) newborns were comparable between the study groups. Labor induction was more common, and the second stage of labor was longer in the vegetarian group. Preterm births (< 32^+0^ and < 37^+0^ gestational weeks) were more common in the control group. No differences were found in the mean umbilical artery pH value, 1- and 5- minutes Apgar scores or the number of newborns transferred to the neonatal intensive care unit and neonatal ward. The proportions of hypertensive disorders in pregnancy did not differ between the groups.

**Conclusion:**

According to our results, a vegetarian diet may be considered safe during pregnancy. It was not associated with an increased risk of pregnancy- or neonatal complications.

## Introduction

Pregnancy is a critical period when the mother requires increased energy supply and amounts of nutrients and micronutrients to ensure healthy gestation and optimal fetal growth and development. Features like energy restriction [1] and lack of important micronutrients [2], [3] possibly disturbing fetal growth may be more common among vegetarians.

There have been contradictory reports concerning the occurrence of low birthweight (LBW) among newborns. In earlier studies vegetarian mothers exhibited an increased risk of delivering an LBW infant [4], [5], [6], [7]. However, the weighted mean neonatal birth weight in five studies suggested no difference between vegetarian and omnivores [8]. In one report five studies reported vegetarian mothers had lower birthweight babies, yet two studies reported higher birthweights [3]. They also reported of increased hypospadias in infants of vegetarian mothers [3].

In one study, compared with omnivorous mothers, vegans had a higher prevalence of preeclampsia [7]. However, a vegetarian diet has been found to be protective against several pregnancy complications associated with disturbances in fetal growth, such as preeclampsia, gestational diabetes (GDM), and preterm delivery [9], [10], [11], [12], [13], [14]. It has also been associated with lower maternal gestational weight gain, in contrast to diets with higher intakes of protein, animal fats, and energy-rich foods, suggesting that plant-based dietary patterns could be beneficial in preventing gestational weight gain and consequently gestational diabetes [6], [15], [16].

As vegetarian diet is becoming increasingly popular [17], [18], [19], the safety of a vegetarian diet during pregnancy has, however, remained unclear [20], [21], [22]. In earlier studies, the results have been conflicting [3], [5], [6], [7], [15], [16], [17], [18], [19]. Our aim was to evaluate the effects of a vegetarian diet on pregnancy outcomes in a Nordic setting. Our hypothesis was that small for gestational age (SGA) and LBW infants would be more common in the vegetarian diet group. In our study birthweight and frequency of SGA were the primary outcomes. Other obstetric parameters concerning pregnancy, labor, and newborn served as secondary outcomes.

## Material and methods

We collected data from the hospital pregnancy and delivery database at Tampere University Hospital. The study period was from January 2015 to April 2021. The registry compiles information on maternal demographic data, pregnancy complications, labor and delivery data, and neonatal health during the first seven days after birth. All pregnant women filled out a questionnaire concerning their diet. For our purposes, a vegetarian diet did not include meat, fish, or poultry, but milk products were allowed. Concomitant gluten free diets were excluded.

In our analysis we expected a 4 % proportion of general population to have SGA newborns. To show a meaningful doubling of SGA cases among vegetarians a sample of 121 vegetarians and 242 control patients would be needed. A total of 150 women on a vegetarian diet were selected from the database during the study period. We opted for one woman following a vegetarian diet and two women following omnivorous diet (n = 300) to constitute the control group. The subjects were according to age and parity.

Maternal, pregnancy, and neonatal outcomes of interest were recorded and compared between these two groups. We evaluated the baseline characteristics of the mothers, including age, body mass index (BMI), parity, smoking during pregnancy, pre-pregnancy diabetes, and infertility treatments. Data concerning pregnancy complications, diagnoses during pregnancy and the characteristics of labor and delivery were gathered, likewise data on the newborns, including birth weight, 1- and 5-minute Apgar scores, umbilical artery pH and possible treatment on a neonatal ward. Maternal anemia during pregnancy was defined as a hemoglobin value below 100 g/l.

GDM was diagnosed with a two-hour oral glucose tolerance test, which is usually done between 24 and 28 weeks of pregnancy for all pregnant women excluding primiparas < 25 years with normal BMI and multiparas < 40 years and normal BMI without previous macrosomic newborn. Oral glucose tolerance test is pathological if even a single value is abnormal or borderline. The Finnish working group recommends diagnostic plasma glucose thresholds of ≥ 5.3 mmol/l (fasting value), ≥ 10.0 mmol/l (1 h), and ≥ 8.6 mmol/l (2 h) for gestational diabetes [23].

Chronic hypertension was defined as systolic blood pressure greater than or equal to 140 mm Hg and/or diastolic blood pressure greater than or equal to 90 mm Hg before pregnancy or before 20 weeks of gestation, the use of antihypertensives before pregnancy, or the persistence of hypertension for more than 12 weeks after delivery [24], [25].

Gestational hypertension was defined as systolic blood pressure greater than or equal to 140 mm Hg and/or diastolic blood pressure greater than or equal to 90 mm Hg after 20 weeks of gestation in a woman who was baseline normotensive [24], [26].

The definition of pre-eclampsia was an increase in blood pressure (systolic blood pressure ≥ 140 mmHg or diastolic blood pressure ≥ 90 mmHg) occurring after the 20th week of pregnancy and proteinuria or, in the absence of proteinuria, at least one of the following: thrombocytopenia, increased liver transaminases, increased creatinine, neurological symptoms (e.g. headache, visual disturbances) or fetal growth retardation [27].

SGA newborns were defined as having a birthweight ≤ 2 standard deviation (SD) or ≤ 10 % below the national sex-specific standard, and newborns who were large for gestational age (LGA) were defined as having a birthweight ≥ 2 SD above it [28].

The Ethics Committee of Tampere University Hospital Finland (R21632) approved the study.

### Statistics

Differences between the omnivores and vegetarian diet groups were tested using Pearson’s Chi-square test, Fisher’s exact test or the Mann–Whitney test. Statistical analyses were carried out using IBM SPSS Statistics version 28.0 software (IBM SPSS, Chicago, Illinois). Two-sided p-values of under 0.05 were considered statistically significant in all analyses.

## Results

The groups did not differ from each other according to maternal characteristics. Table 1.

**Table 1:** Maternal baseline characteristics (N = 450).

	Vegetarian group (n = 150)		Control group (n = 300)		p
Age, years, mean (standard deviation)	30.5	(5.2)	30.4	(5.2)	0.862
BMI, median (range)	25	(22 −29)	24	(22 −28)	0.280
BMI ≥ 30 kg/m^2^, n (%)	26	(17.3)	51	(17.1)	0.954
Primiparity, n (%)	70	(46.7)	140	(46.7)	1.000
Earlier cesarean section, n (%)	18	(12.0)	29	(9.7)	0.446
Smoking during pregnancy, n (%)	28	(18.7)	50	(16.7)	0.359
Pre-pregnancy diabetes mellitus, n (%)	2	(1.3)	5	(1.7)	1.000

Differences between the vegetarian and control groups were tested using independent samples t-tests, the Mann–Whitney test, and Pearson’s Chi-square or Fisher`s Exact test. Missing values (n < 10) were not taken into account.

BMI; body mass index

Table 2 shows data on the newborns. In the vegetarian group, newborns were heavier and taller than in the control group. The frequencies of newborns weighing ≤ 2500 g were significantly lower in the vegetarian group. No real difference could be found between the vegetarian (1.3 % with 95 % Confidence Interval 0 %−3.1 %) and the control group (3.7 % with 95 % CI 1.6 %−5.8 %) according to the percentage of SGA infants (p = 0.235). Instead, the rate of SGA at the 10 % cutoff was lower (p = 0.004) for four newborns in the vegetarian group (2.7 % with 95 % CI 0.9 %−5.2 %) than for 31 newborns in the control group (10.3 % with 95 % CI 6.9 %−13.8 %). LGA proportions did not differ between the vegetarian and the control group (p = 0.179). There were no differences according to 1- or 5-minute Apgar scores and need for neonatal intensive care unit and the neonatal ward.

**Table 2:** Data on the newborns (N = 450).

	Vegetarian group (n = 150)		Control group (n = 300)		P
Birthweight, g, Median (Md), (IQR)	3600	(3298 −3973)	3463	(3026 −3760)	**< 0.001**
Birthweight, g, n (%)					**< 0.001**
< 1500	1	(0.7)	11	(3.7)	
1500–2499	2	(1.3)	23	(7.7)	
2500–3999	113	(75.3)	227	(75.7)	
> 4000	34	(22.7)	39	(13)	
Birthweight in relation to size for gestational age*					0.214
SGA	2	(1.3)	11	(3.7)	
AGA	140	(93.3)	280	(93.3)	
LGA	8	(5.3)	8	(2.7)	
Birth height, cm, Md (IQR)	51.0	(49.0 −52.0)	50.0	(48.0 −51.0)	**0.001**
Apgar scores 1 min, Md (Range)	9	(2 −9)	9	(0 −10)	0.078
< 7, n (%)	13	(8.7)	28	(9.4)	0.825
Apgar scores 5 min, Md (Range)	9	(1 −9)	9	(0 −10)	0.073
< 7, n (%)	12	(8.0)	17	(5.7)	0.347
Umbilical artery pH, Md (IQR)	7.24	(7.17 −7.31)	7.24	(7.18 −7.31)	0.812
Treatment at neonatal intensive care unit and on neonatal ward	27	(18.0)	60	(20.0)	0.613
Ventilator treatment	1	(0.7)	2	(0.7)	1.000
Resuscitation	3	(2.0)	3	(1.0)	0.405
Antibiotic treatment	13	(8.7)	20	(6.7)	0.443

Differences between the vegetarian and control groups were tested using Pearson’s Chi-square test, Fisher`s exact test or Independent-samples Mann-Whitney U-test. *One gestational week was missing in the control group. Md=Median; IQR=Interquartile range

Preterm birth (< 37^+0^ gestational weeks) was more common in the control group, as shown in Table 3. Labor induction was more common in the vegetarian group, but the use of oxytocin was more common in the control group. There were no significant differences in the mode of delivery between the groups, nor was the vacuum extraction rate statistically higher in the vegetarian group (11.3 % with 95 % CI 6.2 %−16.4 %) compared to deliveries in the control group (5.3 % with 95 % CI 2.8 %−7.8 %). The second stage of labor was slightly longer in the vegetarian group.

**Table 3:** Data on deliveries (N = 450).

	Vegetarian group (n = 150)		Control group (n = 300)		p
Gestational weeks, n (%)					**0.024**
22 + 0–31 + 6	1	(0.7)	10	(3.3)	
32 + 0–36 + 6	4	(2.7)	26	(8.7)	
37 + 0–42 + 0	140	(93.3)	256	(85.3)	
> 42	5	(3.3)	7	(2.3)	
Labor induction, n (%)	65	(43.3)	91	(30.3)	**0.006**
Oxytocin, n (%)	60	(40.0)	163	(54.3)	**0.004**
Duration of labor (h:min), Md (IQR)	8:13	(4:49– 13:08)	7:56	(1:15–32:41)	0.613
First stage of labor (h:min), Md (IQR)	7:17	(4:25 −11:22),	7:20	(4:37 −10:24)	0.759
Second stage of labor (min), Md (IQR)	15	(7 −31)	12	(5 −23)	**0.037**
Third stage (min), Md (IQR)	10	(7 −12)	9	(7 −11)	0.175
				n = 251	
Duration of the first stage labor more than 12 h, n (%)	27	(18)	45	(15)	0.714
Mode of delivery, n (%)					0.064
Spontaneous vaginal	102	(68.0)	214	(71.3)	
Vacuum delivery	17	(11.3)	16	(5.3)	
Vaginal breech delivery	1	(0.7)	9	(3.0)	
Elective cesarean section	11	(7.3)	29	(9.7)	
Acute cesarean section	18	(12.0)	32	(10.7)	
Emergency cesarean section	1	(0.7)	0	(0.0)	
Epidural or spinal analgesia, n (%)	87	(58.0)	187	(62.3)	0.375
Perineal rupture grade 3–4, n (%)	2	(1.3)	0	(0.0)	0.111
Manual removal of placenta, n (%)	1	(0.7)	7	(2.3)	0.273
Placental weight g, Md (IQR)	633	(185–980)	610	(207 −2150)	**0.039**
Total bleeding (mL), Md (IQR)	425	(50–2200)	400	(100 −3100)	0.175

Differences between the vegetarian and control groups were tested using Pearson’s Chi-square test, Fisher`s exact test or Independent-samples Mann-Whtiney U-test. Md=Median; IQR=Interquartile range

Table 4 presents the findings during pregnancy. Anemia tended to be more common in the vegetarian group, but it was not common in either group. GDM with or without insulin treatment was more common in the vegetarian group, The proportions of hypertensive disorders in pregnancy did not differ between the groups.

**Table 4:** Pregnancy data (N = 450).

	Vegetarian group (n = 150)		Control group (n = 300)		
	n	(%)	n	(%)	p
Spontaneous pregnancy	139	(92.7)	283	(94.3)	0.490
Infertility treatment	11	(7.3)	17	(5.7)	0.335
Anemia during pregnancy	6	(4.0)	3	(1.0)	0.066
Gestational diabetes	43	(28.7)	58	(19.3)	**0.025**
Insulin-treated gestational diabetes	13	(8.7)	13	(4.3)	0.063
Preeclampsia	3	(2.0)	7	(2.3)	1.000
Gestational hypertension	8	(5.3)	21	(7.0)	0.497
Chronic hypertension	7	(4.7)	4	(1.3)	**0.048**
Cholestasis of pregnancy	0	(0.0)	2	(0.7)	0.555

Differences between the vegetarian and control groups were tested using Pearson’s Chi-Square test or Fisher`s Exact test.

## Discussion

Overall, the pregnancy and neonatal outcomes were favorable in women on a vegetarian diet in our case-control study. In contrast to our hypothesis, a vegetarian diet did not increase the risk of low birthweight or SGA neonates. These women had fewer preterm births, but more GDM compared with omnivorous women.

It has been suggested that a balanced vegetarian diet could be protective against poor pregnancy outcomes such as preeclampsia, GDM, and preterm delivery [10], [11], [15]. Also, in our study, preterm birth was less common in the vegetarian group, and a vegetarian diet seemed to promote being born at a “suitable gestational age” (37^+0^–41^+6^). We observed no differences between the incidences of preeclampsia or gestational hypertension – common causes of SGA and preterm delivery. However, GDM was more common in the vegetarian group. The subjects were of the same age, there was no difference in BMI, and the number of multiparas was similar in both study groups. Thus, it is not clear why GDM with or without insulin treatment was more common in the vegetarian group in our study. It was a surprising finding, since a vegetarian diet is considered beneficial in maintaining good glycemic control in pre-and gestational diabetes. This finding may could emphasize that a vegetarian diet does not automatically control the rise of blood sugar but may contain substances with a high glycemic index e.g. rice and white bread. In immigrants, a vegetarian diet may be rich in ingredients with a high glycemic index, and certain ethnic background may increase the risk of GDM. The more frequent occurrence of GDM in the vegetarian group explained at least some of the findings of higher birthweight and the tendency for more LGA newborns in that group. Labor complications did not differ between groups.

In our study anemia tended to be more common in the vegetarian group, but the number of anemic cases was low in the study material as a whole. We had no direct access to individual patient data, so we cannot give a further description of the anemic cases. Vegetarian diet-based iron is absorbed less effectively than iron from meat [3], and vitamin B₁₂ is found almost exclusively in animal-based foods. Although pregnant vegetarian women are recommended to ensure an adequate intake of iron and vitamin B₁₂ supplements, some of the vegetarian women may have had deficient intake during pregnancy. This emphasizes the necessity of counselling during pregnancy.

Induction of labor was more common in the vegetarian group. Nowadays induction of labor is an increasingly common component of intrapartum care. In Tampere University Hospital the percentage of labor inductions increased by over 10 % from 2015 (24 %) to 2021 (36 %). In GDM, labor induction is common, and this may be one explanation for the frequency of induction. However, it seems that the labors get started well because the use of oxytocin was less common in the vegetarian group and no significant differences were found in the duration of the labor and no clinically significant differences were found separately in the first or second stage of labor.

Contrary to our hypothesis, we found that newborns were heavier and taller, and the number of SGA newborns was lower in the vegetarian group. The women in the vegetarian group probably generally followed a balanced vegetarian diet and received enough energy for fetal development and growth. There are reports that infants born to mothers on well-planned vegetarian diets are born at term and have normal birthweight [29]. A lower incidence of preeclampsia among vegetarians may lead to greater growth, but in our study the incidence of hypertensive pregnancy did not differ between the groups.

The main strengths of this study are the relatively large sample size from a single center and our well-documented obstetric data. Due to the retrospective and register-based study design and diets being self-reported, some inaccuracies cannot be excluded.

## Conclusion

According to our results, a vegetarian diet was not associated with small for gestational age newborns, preterm delivery, or any other pregnancy complications.

## CRediT authorship contribution statement

**Jukka Uotila:** Supervision. **Tiina Luukkaala:** Formal analysis. **Kati Tihtonen:** Supervision. **Johanna Kristiina Reijonen:** Writing – original draft, Investigation.

## Declaration of Competing Interest

None.
